# Composting Reduces the Vitality of H9N2 in Poultry Manure and EMCV in Pig Manure Allowing for an Environmentally Friendly Use of These Animal Wastes: A Preliminary Study

**DOI:** 10.3390/microorganisms8060829

**Published:** 2020-05-31

**Authors:** Kwang-Hwa Jeong, Dong-Jun Lee, Dong-Hyun Lee, Balasubramani Ravindran, Soon Woong Chang, Hupenyu Allan Mupambwa, Myung-Gyu Lee, Hee-Kwon Ahn

**Affiliations:** 1 Department of Animal Environment, National Institute of Animal Science (NIAS), RDA, Wanju 55365, Korea; leedongjun1018@gmail.com (D.-J.L.); andrewlol@korea.kr (D.-H.L.); 2 Department of Environmental Energy and Engineering, Kyonggi University, Youngtong-Gu, Suwon, Gyeonggi-Do 16227, Korea; swchang@kyonggi.ac.kr; 3 Sam Nujoma Marine and Coastal Resources Research Centre, Sam Nujoma Campus, University of Namibia, P. Bag 462 Henties Bay, Namibia; hmupambwa@unam.na; 4 Department of Environmental Engineering, Sangji University, Wonju 26339, Korea; mglee@sangji.ac.kr; 5 Department of Animal Biosystems Science, Chungnam National University, Daejeon 34134, Korea; hkahn@cnu.ac.kr

**Keywords:** microbial inactivation, dialysis cassette, panzootic pathogen, thermophilic, zoonotic

## Abstract

In our study, we monitored the inactivation of two important viruses that are critical in animal husbandry throughout the world. To evaluate the influence of the composting process on inactivation of avian influenza virus (H9N2) in poultry manure compost (PMC) and Encephalomyocarditis virus (EMCV) in pig (swine) manure compost (SMC), the H9N2 and EMCV were injected in dialysis cassettes and buried in two different manure compost piles of poultry and pig manure, respectively. The highest temperature achieved in the PMC and SMC piles during the test period were 75 °C and 73.5 °C, respectively. At the completion of the composting for 168 h, inactivation effect appeared to be more sensitive in H9N2 than EMCV. The vitality of H9N2 decreased by 6.25 ± 0.35 log_10_TCID50/mL to 0.0 log_10_TCID50/mL within 1 h of the composting. The vitality of EMCV decreased from 7.75 ± 0.35 log_10_TCID50/mL to 1.50 log_10_TCID50/mL within 24 h of starting the composting process. However, the activation of EMCV was not decreased (from 7.75 ± 0.35 to 7.50 ± 0.71 log10TCID50/mL) in the control treatment (not inserted in composts) after 168h, while the activation of H9N2 in dialysis cassettes was significantly decreased (from 6.25 ± 0.35 log_10_TCID50/mL to 2.00 ± 0.6 log_10_TCID50/mL). Our study demonstrated the effectiveness of the composting treatment in inactivating the viruses studied, which was not the case with air treatment.

## 1. Introduction

The increase in the global population has resulted in the intensification of industrial and agricultural activities to meet growing human demand for more resources and food [1,2]. In the agricultural industry, global farm animal production is expected to grow faster than any other agricultural sector in the world [3]. Livestock farmers have been trying to increase the number of livestock heads in order to produce more livestock products on their farms, resulting in higher livestock density per unit area.

These practices have led to increased animal manure generation and vulnerability of livestock to diseases carried over by this excreta. There have been cases of mass infection of livestock by malignant pathogens such as avian influenza (AI) [4] and foot and mouth disease (FMD). These pathogens are highly contagious and deadly to poultry and pigs [5,6], and can survive for a considerably long period at room temperature and even under sub-zero conditions [7]. Thus, it is of paramount importance to introduce and use appropriate management approaches to combat and to avoid the potential health risks derived from increasing concentration of livestock production and related concentration of livestock manure within a geographic area [7,8].

Considering the possibility that wild birds and rats can be potential carriers of highly infective pathogenic diseases contained in livestock manure, the use of composting for disposing of and treating manure may help to prevent the spread of such diseases. Since pathogenic viruses are alive in livestock manure compost, there is a potential risk of infectious disease transmission. This raises the importance of evaluating the effect of composting on the vitality of these pathogenic viruses. The encephalomyocarditis virus (EMCV) is a small non-enveloped single-strand RNA virus, and the causative agent of not only myocarditis and encephalitis, but also neurological diseases, reproductive disorders, and diabetes in many mammalian species [9]. The H9N2 virus is a panzootic pathogen that affects poultry causing mild to moderate respiratory distress but has been associated with high morbidity and considerable mortality [10]. In the present study, we evaluated the effectiveness of the composting process on the inactivation of two zoonotic pathogens i.e., H9N2 and EMCV present in poultry and pig manures, respectively.

## 2. Materials and Methods

### 2.1. Feedstock Materials and Experimental Set Up

Pig and poultry manures were collected from farms where no disease occurred. Their physicochemical characterization was done according to Baird et al. [4] and is shown in Table 1 and Table 2.

**Figure 1:**
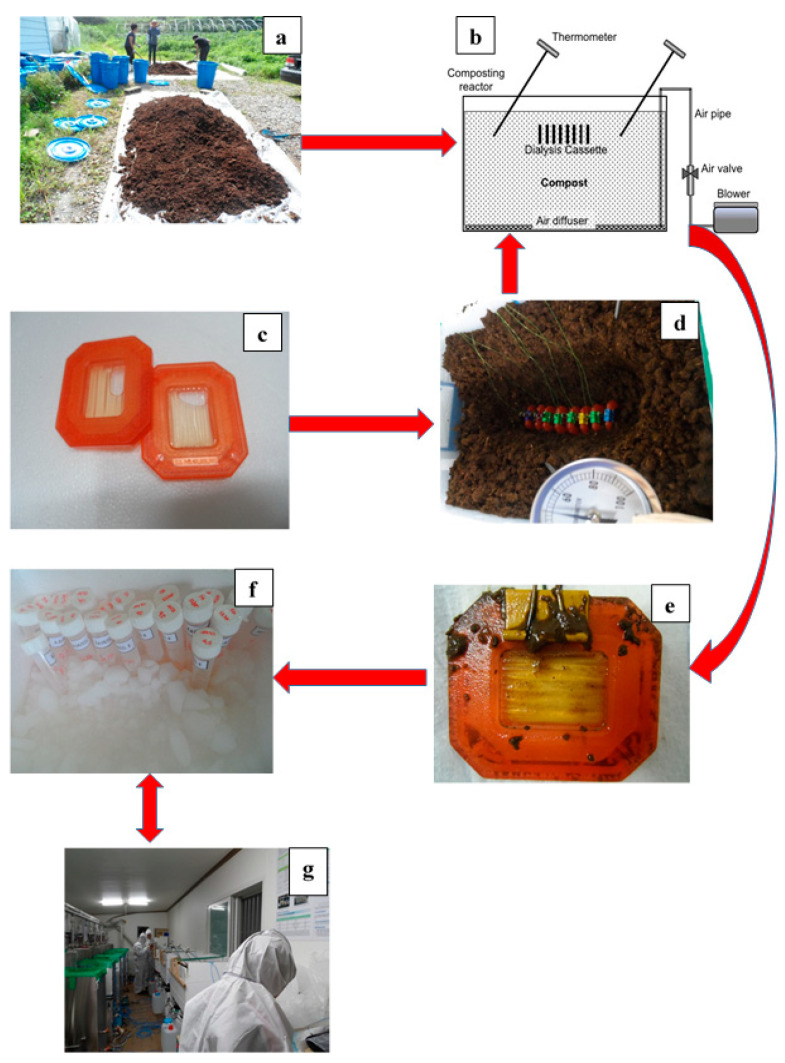
Flow diagram showing the processes involved in experimental set-up and sample collection in our study. (**a**) compost preparation. (**b**) Schematic diagram of composting reactor. (**c**) Cassette before burial in the compost. (**d**) Dialysis cassettes containing virus (H9N2, EMCV) buried in compost. (**e**) Cassette recovered from the compost. (**f**) Virus samples collected from dialysis cassettes recovered from compost pile. (**g**) Laboratory for composting experiment.

The study consisted of five treatments namely pig manure with aeration, poultry manure with aeration, pig manure without aeration, poultry manure without aeration, and a control without manure. For the manure-based treatments, pig and poultry manures were initially mixed with sawdust as a bulking material, and cured compost was added as an inoculator at a volumetric ratio of 7:2:1, respectively (Figure 1). For each experimental unit, mixing of the manures, saw dust and composts was done separately. The study was laid in a completely randomized design with three replicates for each treatment.

For the experimental set up, rectangular composting reactors with a volume of 150 L were used and placed in an indoor laboratory under controlled conditions. The laboratory was equipped with air conditioning facilities to maintain a constant temperature of 25 °C. Some of the reactors were equipped with air diffusers at the bottom for air supply to the compost pile. In these reactors, air was supplied at a rate of 1.2~1.4 m^3^ min^−1^ per m^3^ of compost using an air flow controller installed in the piping section as illustrated in Figure 1. The outside of the composting reactor was covered with expended polystyrene with a thickness of 3 cm to minimize the influence of outside air conditions. However, though insulation was provided by the polystyrene used in our study, its R value (thermal resistance) was not originally established. The various physico-chemical properties of the different treatments were undertaken using standard procedures [1,2].

### 2.2. Virus Vitality Test Process

Since H9N2 and EMCV viruses should be cultured and analyzed in a government-licensed laboratory, they were cultivated in a veterinary secure laboratory and then moved to the composting experimental site. After injecting the virus into dialysis cassettes (Slide-A-Lyzer Dialysis Cassettes; Thermo Scientific, USA), these cassettes were then buried into the different compost piles as illustrated in Figure 1 and previously shown by Glanville et al. [11] for AI virus inactivation test. For the control treatments, the dialysis cassettes were injected with virus culture medium, and placed in the same room under the same environmental conditions where the composting trial took place. Enough cassettes were incubated in each treatment to allow for destructive sampling with 3 replications per sampling time. The dialysis cassettes injected with virus culture medium were then randomly collected at 1, 3, 6, 12, 24, 48, 84, and 168 h. The solution containing the virus in the collected dialysis cassette was transferred into a sample storage container at the temperature of below zero and analyzed for virus activation in a secure laboratory (Figure 1). For control experiments, the virus-injected cassettes were placed in a mesh bag installed in the air at a height of 1.5 m from the bottom of the composting laboratory, and recovered at the same time as the experimental cassettes of the composting group to analyze the vitality change of H9N2 and EMCV in the atmosphere.

### 2.3. Virus Vitality Post Treatment Analysis

Briefly, for preparing H9N2 influenza virus (10^7^ TCID50/mL) cultured in MDCK cells, gentamicin 500 μg uL^−1^ was added to each sample solution and incubated at room temperature for 1 h. Centrifugation process (13,000 rpm/18,000× *g* for 5 min) was carried out to remove contaminants from the sample. The supernatant was diluted and a 25 μL of diluted sample was distributed to 96 well cell culture plate according to dilution drainage. A 175 μL of minimum essential media (MEM; including antibiotics) was added to each well of 96 well cell culture plates containing samples and incubation process was performed (37 °C for 3 days). After 3 days, microscopic observations to find specific cytopathic effect (CPE) of influenza virus was carried out and the sample with CPE-identified was used to titer for the virus [12]. Briefly, for preparing EMCV (10^8^ TCID50/mL) cultured in Vero cells, gentamicin (500 μg μL^−1^) was added to each sample solution and incubated at room temperature for 1 h. The samples were centrifuged at 13,000 rpm for 5 min to remove contaminants from the samples. The supernatant was diluted and a 25 μL of diluted sample was distributed to 96 well cell culture plates according to dilution drainage. After that, a 175 Ul of MEM (including antibiotics) was added to each well of 96 well cell culture plate containing samples. After 3 days of incubating at 37 °C, microscopic observations to find specific CPE of EMCV was carried out. Finally, the sample with CPE-identified was used to titer for the virus [12].

## 3. Results and Discussion

### Changes in Selected Physicochemical Properties during Composting

An overview of the physicochemical properties of the two animal manures is shown in Table 1 and Table 2. For the poultry manure composts, moisture content after the 184 h of composting was reduced by 1.78% and 1.65% (*p* > 0.05) under the treatments with and without mechanical aeration, respectively. Similarly, for the pig manure compost, the moisture content reduction was slightly greater under the air blown treatment than in the non-blown treatment, with a 2.44% and 2.35% reduction, respectively (Table 2). Changes in organic matter content during composting are critical in indicating microbial activity and efficiency of decomposition in a compost [13]. In our study, the composts with mechanical aeration resulted in slightly higher changes in organic matter relative to the non-blown composts. On average across the two manures, mechanical aeration resulted in a 1.8% while non-blown treatments resulted in a 0.7% change in organic matter. This could indicate that the air blown treatments resulted in more oxygen being available for microbial activity, hence the enhanced decrease in organic matter observed even after 184 h only. It was noteworthy that there were no changes observed in total nitrogen in all treatments.

**Figure 2:**
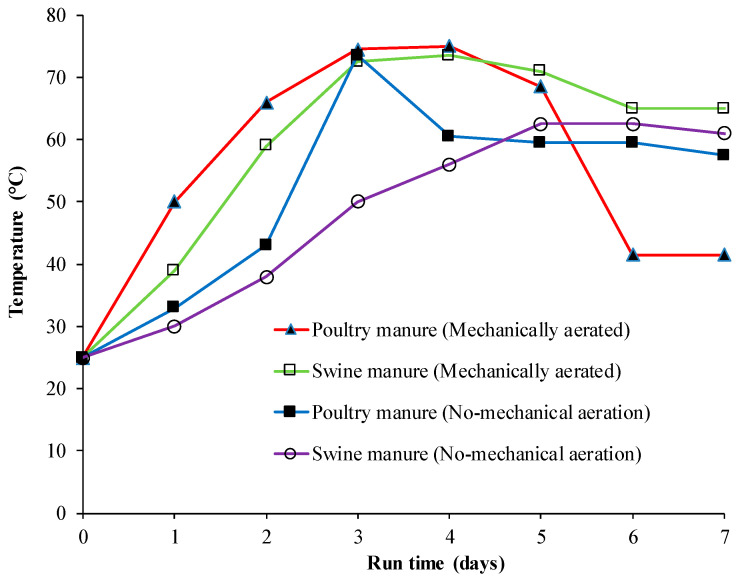
Temperature changes during composting of poultry and pig (swine) manure with or without mechanical aeration.

The temperature changes of the poultry manure and pig (swine) manure composts during the test period are shown in Figure 2.

The temperature started to rise from the beginning of composting process reaching a maximum value between day 3 and 4, which could coincide with the peak of microbial activity. During composting period, the highest temperature of poultry manure and pig manure composts were up to 77.0 °C and 73.5 °C, respectively. This is consistent with results reported by Chang et al. [14], with these high temperatures being attributed to the pathogen kill observed in most thermophilic composts. High temperature of compost is very effective in suppressing the vitality of H9N2 and EMCV [15]. Most viruses rapidly lose their vitality at high temperature. According to the results of a virus inactivation study conducted by Elving et al. [8], both the H7N1 HPAIV strain and the ϕ6 phage were inactivated to levels below the detectable limits within 24 h from the start of the compost trial, with peak temperatures between 42 °C and 67 °C. In our study, in both composts, the temperature increase of compost pile was higher in the treatments with mechanical aeration treatment relative to the non-mechanically aerated treatments, which is most likely linked to the higher microbial activity in the compost pile with more oxygen present.

**Figure 3:**
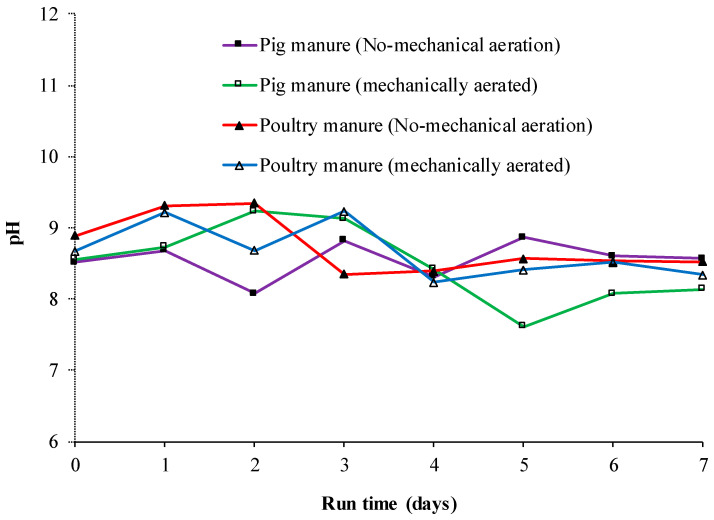
Changes of pH during composting of poultry and pig (swine) manure with or without mechanical aeration.

The pH variation of poultry and pig manure composts during the study period is shown in Figure 3. There was no significant difference in pH across the treatments during the 184 h of composting. Moreover, pH was on the alkaline side and this could be due to accumulation of ammonia during these first stages of composting.

Table 3 shows inactivation effect of H9N2 by the poultry manure composting process. Since H9N2 was injected in poultry manure pile by the MDCK cells, the inactivation effect was observed for 168 h. Within 1 h from commencement of manure composting, the vitality of H9N2 was reduced from 6.25 ± 0.35 log_10_ TCID50/mL to 0 log_10_ TCID50/mL, resulting in 100% killing of the virus (Table 3). 

It was noteworthy that there was no difference in the degree of viral death between the mechanical aeration treatment and the non- mechanically aerated. Similar results were observed by Elving et al. [8], where a highly pathogenic avian influenza virus H7N1 was inactivated at both mesophilic and thermophilic stage. This could also explain the observation that even within the control, the H9N2 vitality was greatly reduced by changing the virus activity from 6.25 ± 0.35 log_10_ TCID50/mL to 2.0 log_10_ TCID50/mL at 168 h after the start of the test. Furthermore, the observation where the vitality decreased within 1 h of composting also indicate that not only temperature is responsible for this observation but also compost chemical properties.

The test results of the evaluation of the inactivation effect of EMCV due to pig manure composting are shown in Table 4. The inactivation effect on EMCV was observed for 168 h. The film of dialysis cassette was damaged 24 h after composting, which was due to exposure to high temperature and gas in compost pile. The vitality of the EMCV in swine manure compost showed a sharp decrease from 1 h after the start of the composting. The EMCV of the samples in SMC was decreased from 7.75 ± 0.35 log_10_TCID50/mL at the start to 1.50 log_10_TCID50/mL after 24 h of composting treatment (Table 4). Bøtner [16] in a similar study reported the temperature dependent inactivation of Aujeszky’s Disease Virus (ADV) during aerobic storage at temperatures at 5 °C and 55 °C. In this study, inactivation of virus took approximately 15 weeks under the control of 5 °C, while no virus was detected after 10 min at 55 °C. Viral deactivation in swine manure compost was decreased within 24 h after the start of the composting, and the rate of reduction reached 99.99%. According to the measurement of the degree of inactivation of the EMCV of the samples buried in pig manure compost, the time required for inactivation of EMCV was shorter from the mechanically aerated treatment than the non-mechanically aerated treatment composting reactor. Therefore, air supplying to compost pile appears to be an effective practice for inactivating EMCV in manure composting process [17]. The vitality of EMCV in the control (the sample left in the air) was changed from 7.75 ± 0.35 log_10_TCID50/mL (at the start of the test–time 0) to 7.50 log_10_TCID50/mL (at the end of the test- time 168 h).

## 4. Conclusions

Manure composting practice has been identified to have potential in the H9N2 and EMCV inactivation. The inactivation effect of H9N2 and EMCV was relatively good when air was blown into the livestock manure compost pile. However, the H9N2 and EMCV left in the air (control) appeared to maintain their vitality even after the experimental periods, so the effect of composting on the vitality of H9N2 and EMCV seem to be effective. This study therefore provides preliminary evidence that the vitality of H9N2 and EMCV that cause infectious diseases in livestock manure is significantly lowered by composting.

## Figures and Tables

**Table 1:** Characteristics of poultry manure used in this study.

Classification	Poultry Manure Compost (Initial)		Poultry Manure Compost (Final)	
Treatment	Mechanically Aerated	Non-Mechanically Aerated	Mechanically Aerated	Non-Mechanically Aerated
Water content (%)	63.38	62.27	62.25	61.24
Organic matter (%)	28.57	29.85	27.98	29.83
OM/N	18.19	19.38	18.28	19.24
TN (%)	1.57	1.54	1.53	1.55

**Table 2:** Characteristics of pig manure used in this study.

Classification	Swine Manure Compost (Initial)		Swine Manure Compost (Final)	
Treatment	Mechanically Aerated	Non-Mechanically Aerated	Mechanically Aerated	Non-Mechanically Aerated
Water content (%)	60.75	60.54	59.27	59.12
Organic matter (%)	29.10	30.00	28.67	29.58
OM/N	25.30	25.21	25.89	25.00
TN (%)	1.15	1.19	1.12	1.20

**Table 3:** Change of vitality of H9N2 with time during composting of poultry manure.

Classification	Vitality of H9N2 (log 10 TCID_50_/_mL_)		
	Control	Poultry Manure Compost (Mechanically Aerated)	poultry Manure Compost (No Mechanical Aeration)
Start	6.25 ± 0.35	6.25 ± 0.35	6.25 ± 0.35
1 h	7.00 ± 0.42	0	0
3 h	6.75 ± 0.35	0	0
6 h	6.25 ± 0.35	0	0
12 h	6.00 ± 0.21	0	0
24 h	5.75 ± 0.35	0	0
48 h	6.00 ± 0.71	0	0
84 h	5.50 ± 0.23	-	0
168 h	2.00 ± 0.6	-	-

Note: Means ± standard deviation.

**Table 4:** Change of vitality of encephalomyocarditis virus (EMCV) with time during composting of pig (swine) manure.

Classification	Vitality of EMCV (log _10_ TCID_50_/_mL_)		
	Control	Pig Manure Compost (Mechanically Aerated)	Pig Manure Compost (No Mechanical Aeration)
Start	7.75 ± 0.35	7.75 ± 0.35	7.75 ± 0.35
1 h	7.25 ± 0.35	3.50 ± 2.83	6.50 ± 0.29
3 h	7.25 ± 1.06	1.50 ± 0.25	2.00 ± 0.15
6 h	7.25 ± 0.35	1.75 ± 0.35	2.00 ± 0.71
12 h	7.00 ± 0.98	2.00 ± 0.71	2.00 ± 0.71
24 h	7.75 ± 0.35	1.50 ± 0.21	1.50 ± 0.24
48 h	7.50 ± 0.71	-	-
84 h	7.00 ± 0.56	-	-
168 h	7.50 ± 0.71	-	-

Note: Means ± standard deviation.
